# Supplementary material for: Chromothripsis during telomere crisis is independent of NHEJ, and consistent with a replicative origin
Source: Genome Res. 2019 May;29(5):737–49. doi: 10.1101/gr.240705.118 (PMC6499312; doi:10.1101/gr.240705.118)
Supplement: Supplemental Material [file supp_gr.240705.118_Supplemental_file_1.zip › contigs/annotated_contigs/DB79/contig.3.DB79_length_388_mean_cov_8.7293814433.docx]

**DB79_length_388_mean_cov_8.7293814433**

```
CCTGACAAACCAAATGAATGACAAGGGCAGAACCAGAATTCTCACTCACATCCCCAAATGTGGCAAGAGGAAGTCTTTTTCCTCTTCAG
 >chr8:22254072-22254357 - E=2e-146
TTGAGTAGTAGGAGGAAAAGCTACCGGGGAAGAACAAAAAAAAAACGGTGCACCTGGACCAAAAACAATGGGCTGACTCCCTTGGAAGG

AAATAAGGGCTCCCGGGCCACATTCTGAATTGAGGACATGATCCAATTCCAAATTCCACTTAGTCGTCCTTCTCTCTCTCTCTCTCTCT

CTCTCTCTCTCTCTCTCTCTC|CGCCCCC|CCCCTCTCTATTTATTTATTTATTTTTTA|A|GATGGAGTCTTGCTCTGTTGCCCAGGC
                             >chr3:73371882-73371912 + E=2e-03 >chr6:89181864-8918192
TGGAGTGCAGTGGCGTGATCTCGGCTCACCGTAACC8 - E=5e-21
```
